# *Brucella* infection and Toll-like receptors

**DOI:** 10.3389/fcimb.2024.1342684

**Published:** 2024-03-12

**Authors:** Hui Yu, Xinyi Gu, Danfeng Wang, Zhanli Wang

**Affiliations:** ^1^ Inner Mongolia Key Laboratory of Disease-Related Biomarkers, The Second Affiliated Hospital, Baotou Medical College, Baotou, China; ^2^ School of Basic Medicine, Baotou Medical College, Baotou, China; ^3^ The College of Medical Technology, Shanghai University of Medicine & Health Sciences, Shanghai, China

**Keywords:** *Brucella*, Toll-like receptor, innate immunity, pathogen-associated molecular pattern, vaccine

## Abstract

*Brucella* consists of gram-negative bacteria that have the ability to invade and replicate in professional and non-professional phagocytes, and its prolonged persistence in the host leads to brucellosis, a serious zoonosis. Toll-like receptors (TLRs) are the best-known sensors of microorganisms implicated in the regulation of innate and adaptive immunity. In particular, TLRs are transmembrane proteins with a typical structure of an extracellular leucine-rich repeat (LRR) region and an intracellular Toll/interleukin-1 receptor (TIR) domain. In this review, we discuss *Brucella* infection and the aspects of host immune responses induced by pathogens. Furthermore, we summarize the roles of TLRs in *Brucella* infection, with substantial emphasis on the molecular insights into its mechanisms of action.

## 1 Introduction

Brucellosis is a classical bacterial zoonosis found worldwide (de Figueiredo et al., 2015). Based on clinical presentation, brucellosis can be classified as acute, subacute, or chronic. Although the mortality rate of brucellosis is low, it can cause chronic complications or even disabilities, which severely endanger human health (Willems et al., 2022).

Brucellosis is caused by *Brucella* infection. *Brucella*, a facultative intracellular pathogen belonging to the phylum Proteobacteria, was discovered by David Bruce in 1887 (Głowacka et al., 2018). Originally, *Brucella* was thought to contain three species: *Brucella abortus*, *Brucella melitensis*, and *Brucella suis*. Presently, more species have been identified in both domesticated and wildlife species, such as *Brucella ceti*, *Brucella canis*, *Brucella ovis*, *Brucella neotomae*, and *Brucella microti*. It has been reported that some *Brucella* species can be further classified into biovars. For example, *B. abortus* has nine biovars, and *B. suis* contains five biovars (Boschiroli et al., 2001).

*Brucella* contains various virulence factors. The type IV secretion system (T4SS) is highly conserved among *Brucella* species and is a major virulence factor. Additionally, lipopolysaccharide (LPS) is identified as a key pathogenicity determinant of *Brucella*. Furthermore, previous reports have claimed that *Brucella* virulence factor A (BvfA), base excision repair (BER), and BvrR/BvrS system are also essential for virulence (Boschiroli et al., 2001). It has been proven that virulence factors play key roles in chronic persistence of *Brucella*.

## 2 *Brucella* infection

*Brucella* can infect the host by inhalation or ingestion, through genital mucosa, and even via injured skin. In general, *Brucella* rapidly translocates in the mucosal epithelium to the body, where it is efficiently endocytosed by main target cells including mucosal macrophages and dendritic cells (DCs) and subsequently invades and survives within specialized phagocytes (such as macrophages) instead of non-specialized phagocytes (such as epithelial cells), circumventing and modulating the host’s immune responses (Dominguez-Flores et al., 2023). Recently, researchers investigated the molecular mechanisms of chronic *Brucella* infections using non-specialized phagocyte HeLa cells. The results showed that *Brucella* was able to inhibit phagosome–lysosome fusion and replicate in a different compartment (Kim et al., 2004).

Four steps must be taken for *Brucella* to infect a host: adhesion, cellular internalization, intracellular growth, and transmission (Parihar et al., 2019). A number of adhesins have been identified in *Brucella*, such as the bacterial Ig-like (Blg-like) domain-containing proteins, the monomeric autotransporters, Bp26, the trimeric autotransporters, the sialic acid-binding proteins, and T4SS-VirB5 (Bialer et al., 2020). These bacterial cell surface proteins enable *Brucella* to bind to host cell surfaces. Once *Brucella* binds to the cell surface, it can be tested by adhering to a variety of cellular receptors. Fc gamma receptor IIa (Hashemi et al., 2007) and complement receptor 3 (CR3) (Gamazo et al., 2006), the conditioning receptors, recognize O-chain fragments of *Brucella* LPS. Non-conditioning receptors include the class A scavenger receptor (SR-A) and Toll-like receptors (TLRs). Compared to conditioning receptors, these receptors have a wide spectrum of biological roles because of their broad ligand-binding capacity. SR-A recognizes lipid A LPS (Kim et al., 2004). TLR2, TLR4, and TLR6 detect *Brucella* LPS and lipoproteins, whereas TLR3, TLR7, and TLR9 can recognize nucleic acid motifs (Oliveira et al., 2008). After detecting *Brucella* through the specific receptors, the host cell activates a signaling pathway that leads to the polymerization of actin filaments (Kusumawati et al., 2000). *Brucella* recognizes lipid rafts contained in the host cell membrane, contributing to the intracellular transport of *Brucella*.

**Figure 1:**
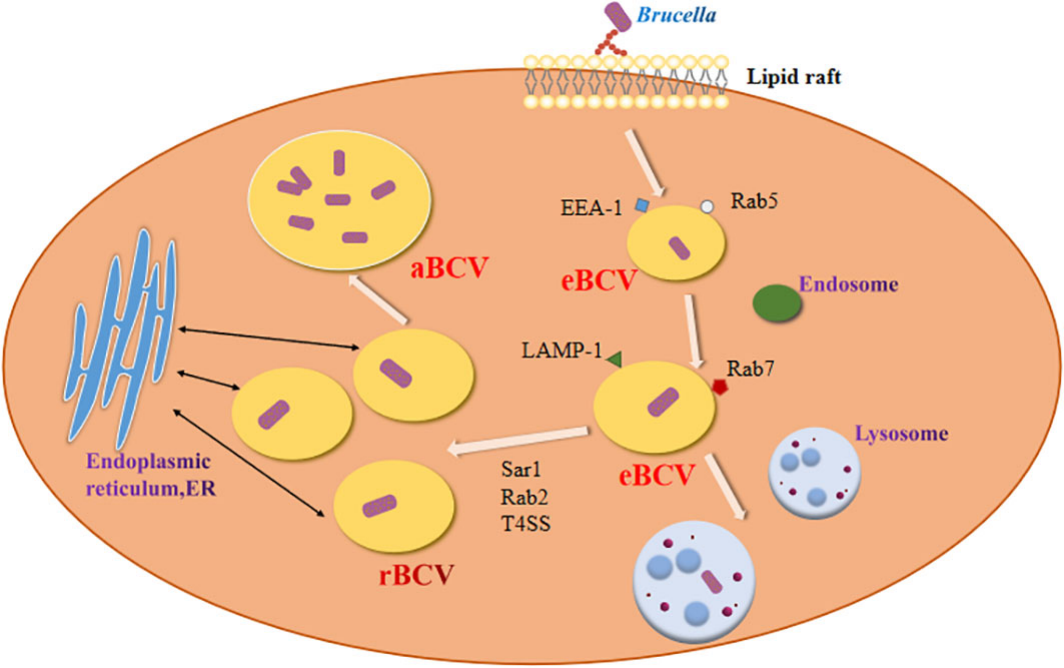
Establishment of *Brucella* infection.

*Brucella* enters host macrophages and forms replicative phagosomes called *Brucella*-containing vacuole (BCV) (**Figure 1**). Early BCV was defined as endosome-like BCV (eBCV), which obtains several marker molecules of the host. As eBCV matures, eBCV loses early endosomal markers and acquires late endosomal and lysosome-recognized marker molecules, which facilitates the fusion of eBCV with lysosomes (Celli, 2019). A proportion of the eBCV escapes lysosomal degradation and reaches the endoplasmic reticulum (ER) (de Bolle et al., 2012), where it then fuses with the ER to generate a replication-permissive BCV (rBCV) (Miller et al., 2017). *Brucella* then proliferates in rBCV. During the late period of bacterial infection, rBCV contains large amounts of *Brucella* and converts to autophagic BCV (aBCV) (Starr et al., 2012). Then, aBCV releases pathogens by both cleavage and non-cleavage mechanisms, and the *Brucella* intracellular life cycle ends (Smith et al., 2016).

## 3 *Brucella* infection elicits immune responses in the host

*Brucella* infection activates innate immunity and subsequently leads to the activation of adaptive immunity (Skendros and Boura, 2013). The innate immune response system constitutes the body’s first line of defense mechanisms that protect the host from pathogen invasion. Innate immune sensing of *Brucella* via pattern-recognition receptors (PRRs) that recognize specific molecular motifs called pathogen-associated molecular patterns (PAMPs) (Skendros et al., 2011; Sadeghi et al., 2023). After PRR recognition of the specific PAMP, some PRRs trigger intracellular signaling in the antigen-presenting cells (macrophages and DCs) and elicit an inflammatory response that effectively destroys the invading pathogen (Golding et al., 2001). The innate immune response against *Brucella* involves PRRs, such as TLRs and Nod-like receptors (NLRs) (Cerqueira et al., 2018). Among the functionally unique class of PRRs, TLRs are optimally characterized and have been intensively studied for their ability to specifically recognize distinct PAMPs from different pathogens (Sadeghi et al., 2023). Recognition of PAMPs by TLRs initiates multiple intracellular signaling cascades mediated by adapter molecules, such as myeloid differentiation factor 88 (MyD88) and Toll/interleukin-1 receptor (TIR) domain-containing adapter-inducing interferon-beta (IFN-beta) (TRIF), leading to the activation of inflammatory factors and the upregulation of co-stimulatory molecules (Sadeghi et al., 2023). For example, TLRs (except TLR3) bind to MyD88 and initiate cellular signaling, resulting in the activation of the IκB kinase complex and p38 mitogen-activated protein kinase (MAPK) (Gomes et al., 2012; Dimitrakopoulos et al., 2013). These pathways lead to the activation of the transcription factors NF-κB (Hop et al., 2017; Peng et al., 2021) and AP-1 (Jiménez de Bagüés et al., 2005), leading to the secretion of proinflammatory cytokines (Baldi and Giambartolomei, 2013). Common PAMPs include numerous microbial products, such as bacterial LPS, peptidoglycan, lipoproteins, flagellin, and nucleic acids (Kumar et al., 2011).

The cell-mediated immune response is the body’s predominant way of fighting *Brucella* (Ali et al., 2023). This includes the activation of antigen-presenting cells such as macrophages and DCs. Macrophages are the frontline cells for defense against Brucella (Jacob et al., 2016), and they play a critical role in innate immunity by phagocytosis and degradation of invading microorganisms (Oliveira et al., 1998). DCs are recognized as one of the most important antigen-presenting cells for eliciting effective cellular immunity. Immature DCs capture and process antigens before migrating to secondary lymphoid organs and presenting specific major histocompatibility complex (MHC) antigens. Upon maturation, DCs express high levels of MHC and costimulatory molecules and improve antigen presentation. Cytokines produced by mature DCs enhance adaptive immunity (Billard et al., 2007; Fabrik et al., 2013; Avila-Calderón et al., 2020).

TLRs play a key role in linking pathogen recognition with the activation of innate immune response and are also essential for initiating the adaptive immune response (Surendran et al., 2012). TLR stimulation can activate the innate immune response through activation of NK cells, DCs, or macrophages and secretion of IFN-α, IFN-γ, and TNF-α (Baldwin and Winter, 1994; Huang et al., 2005; de Almeida et al., 2013). TLR stimulation can also activate adaptive immune responses by promoting cross-presentation, Th1 polarization, and induction of cytotoxic T cells (Arias et al., 2017; Bin Park et al., 2023). There is evidence that the immune responses against most pathogens require multiple TLRs rather than a single TLR (Murugan et al., 2023).

**Figure 2:**
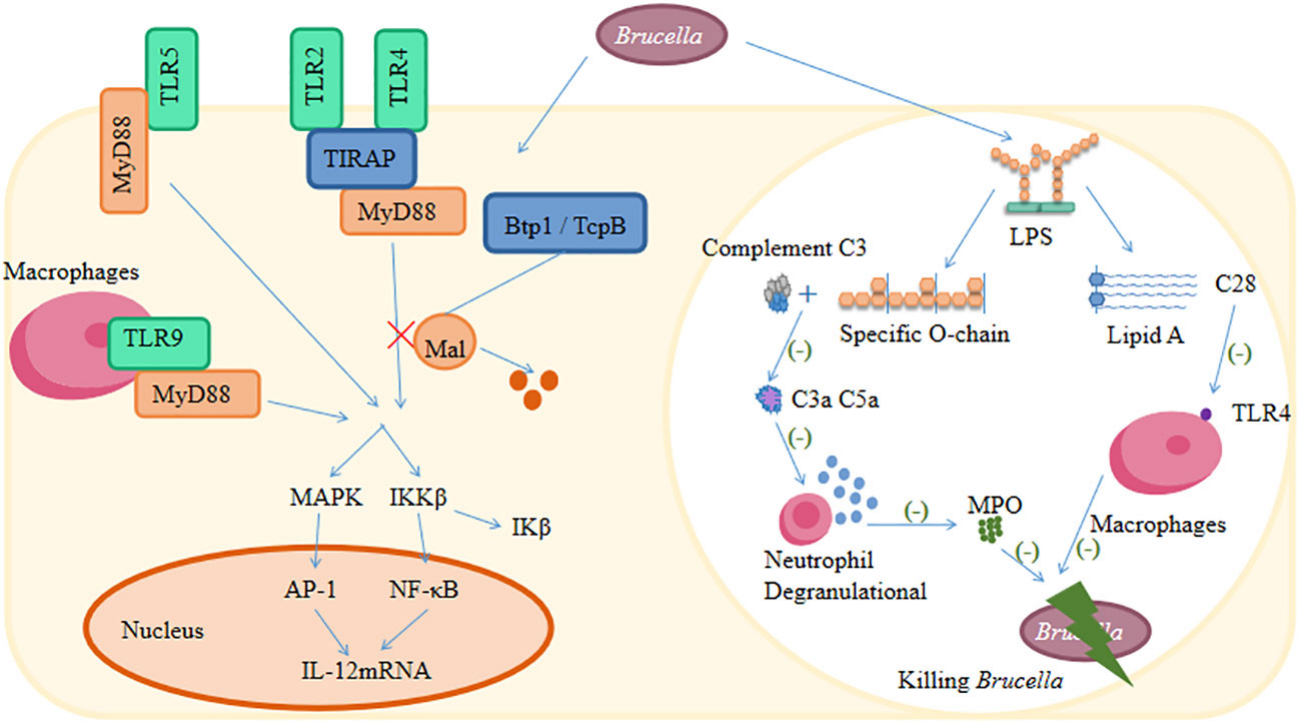
The main escape mechanisms of *Brucella* against the host immune system.

It is well known that *Brucella* has developed a wide range of strategies to evade both innate and adaptive immune responses (Martirosyan and Gorvel, 2013; Skendros and Boura, 2013; Jiao et al., 2021). The main escape mechanisms of *Brucella* against the host immune system are inhibition of the complement pathway and TLR signaling pathways, interference with antigen presentation, selective subversion of the autophagy pathway, inhibition of DC stimulation, inhibition of autophagic lysosomal fusion, and macrophage apoptosis (**Figure 2**) (Radhakrishnan and Splitter, 2010; Rana et al., 2013; Barrionuevo and Giambartolomei, 2019; Stranahan and Arenas-Gamboa, 2021). There are strategies for modifying the LPS to evade effective recognition by TLR4 strategies (Barquero-Calvo et al., 2015; Matamoros-Recio et al., 2023). Additionally, there are also strategies to encode various outer membrane proteins to facilitate their invasion and immunomodulation (Barrionuevo et al., 2008; Velásquez et al., 2017; Pasquevich et al., 2019). Moreover, microRNAs (miRNAs) have recently been found to play a crucial role in immune evasion mechanisms in brucellosis (Ahmed et al., 2016).

## 4 *Brucella* vaccines for humans

A limited number of antibiotics are effective against *Brucella* because of the intracellular lifestyle of these organisms. Therefore, there is a desire to develop a safe and efficacious vaccine for human brucellosis. In order to develop *Brucella* vaccines for humans, an understanding of the mechanisms of the adaptive immune response in brucellosis is required. In fact, CD4+, CD8+, and γδ T cells are stimulated to produce IFN-γ to reduce the intracellular survival of *Brucella*. Additionally, CD8+ and γδ T cytotoxic cells were capable of killing infected macrophages. Furthermore, Th1-type immune response occurs to promote phagocytosis of *Brucella* (Perkins et al., 2010). Several decades ago, various *Brucella* vaccines for humans have been studied, such as *B. abortus* S19 (Vershilova, 1961), *B. melitensis* Rev.1 (Spink et al., 1962), *B. abortus* strain 19BA, and *B. melitensis* 104M (Perkins et al., 2010). However, these vaccines were highly reactogenic or caused brucellosis in humans and were considered unsuitable for human vaccination. Up to now, several human brucellosis vaccine candidates have been reported, including live attenuated vaccines, subunit vaccines, recombinant protein-based vaccines, vectored vaccines, and DNA vaccines (Perkins et al., 2010). An ideal vaccine for use in humans would be considered for its safety, immunogenicity, and protective efficacy (Hashemzadeh et al., 2023). It is well known that the inclusion of adjuvants within vaccines can enhance vaccine-induced protection and thus offer an alternative approach to vaccine development. Interestingly, several TLRLs are now being selectively investigated for the development of novel vaccine adjuvants within vaccines (Duthie et al., 2011).

## 5 Role of TLRs in *Brucella* infection

TLRs are essential for activating the innate immune response and initiating adaptive immunity (Fitzgerald and Kagan, 2020; Duan et al., 2022). TLRs are characterized by sharing an extracellular leucine-rich repeat (LRR) region and an intracellular TIR structural domain (Kawasaki and Kawai, 2014; Kornilov et al., 2023). The LRR structural domain is responsible for binding to PAMP, and the TIR structural domain is responsible for binding to TIR structural domains containing adapter molecules, including MyD88, TIR domain-containing adapter (TIRAP), TRIF, TRIF-related adapter molecule (TRAM), and sterile alpha and HEAT-Armadillo motifs (SARM), to initiate signaling (Brikos and O'Neill, 2008; Cui et al., 2014). MyD88 and TRIF can bind to the TLR, while TIRAP and TRAM act as bridging adapters, binding to MyD88 and TRIF, respectively (Takeda and Akira, 2004; West et al., 2006; Nilsen et al., 2023). SARM can only act as a negative regulator by interacting with TRIF (Carty and Bowie, 2019; Wang et al., 2021). The TLR is localized to the plasma membrane or endocytosis membranes. PAMP recognition of TLRs can initiate signaling and activate transcription factors, which in turn trigger the production of cytokines, chemokines, and antimicrobial peptides, ultimately controlling or clearing the infection (Chen et al., 2005; Sanjeewa et al., 2020).

Of the 13 TLRs identified in mammals (Vijay, 2018), TLR1 to TLR9 are conserved between humans and mice (Kumar and Barrett, 2022). TLR1, 2, 4, 5, and 6 are expressed in cell membranes. TLR3, 7, 8, and 9 are present in the endosomes of cells (Luchner et al., 2021; Fore et al., 2022). TLR2 recognizes a variety of microbial molecules such as peptidoglycan and lipoproteins (Venkataranganayaka Abhilasha and Kedihithlu Marathe, 2021), TLR4 recognizes LPS and several viral envelope proteins (Wu et al., 2022), TLR5 recognizes the flagellum (Clasen et al., 2023), and TLR3, 7, 8, and 9 recognize microbial and viral nucleic acid motifs (Eisenächer et al., 2007). Of these, TLR3 recognizes double-stranded RNA (Sakaniwa et al., 2023), TLR7/8 recognizes single-stranded RNA (Yu et al., 2012), and TLR9 recognizes CpG-DNA (Yu et al., 2017; Takano et al., 2023).

### 5.1 TLR2

The interaction of *Brucella* strains with TLR2 on host cells affects the induction of innate immune responses during infection (Giambartolomei et al., 2004; Macedo et al., 2008; Zwerdling et al., 2008; Pei et al., 2012; Surendran et al., 2012; Lee, K. M. et al., 2013; Weinhold et al., 2013; Ferrero et al., 2014; Arias et al., 2017; Dominguez-Flores et al., 2023). TLR2, located on the cell surface, is required for the production of tumor necrosis factor (TNF) (Huang et al., 2003; Delpino et al., 2012). Previous studies also showed that TLR2 is required for TNF production and regulates TLR9 signaling for the effective induction of IL-12 upon stimulation by heat-killed *B. abortus* (Zhang et al., 2012). In addition, recombinant *Brucella* cell-surface protein 31 (rBCSP31), an agonist of TLR2, induces cytokine production, upregulates macrophage function, and induces a Th1 immune response (Li et al., 2014). *B. abortus* Mdh enhanced Th2-related responses triggered by the MyD88-dependent TLR2 signaling pathway and could induce an inflammatory response in microfold cells (Shim et al., 2020). *B. abortus*-activated microglia induce neuronal death via *Brucella* lipoprotein-mediated TLR2 activation (Rodríguez et al., 2017).

*Brucella* uses various stealthy strategies to avoid activation of the innate immune system (Barquero-Calvo et al., 2007). Researchers identified a new *Brucella* protein Btp1, which down-modulates maturation of infected DCs by interfering with the TLR2 signaling pathway (Salcedo et al., 2008). Similarly, BtpB inhibits TLR2 and disrupts NLRP3 signaling pathways to inhibit host immune responses in early *Brucella* infections (Li, J. et al., 2022). Moreover, *Brucella* encodes a TIR domain-containing protein (TcpB) that mimics the properties of the TLR adaptor protein TIRAP to subvert TLR signaling (Radhakrishnan et al., 2009; Radhakrishnan and Splitter, 2010; Alaidarous et al., 2013; Jakka et al., 2017). Outer membrane vesicles (OMVs) from *B. abortus* also inhibit the cytokine response of monocytes to TLR2 agonists that favor the persistence of *Brucella* within host cells (Pollak et al., 2012). *B. abortus* utilizes its lipoproteins to inhibit IFN-γ-induced expression of the type I receptor for the Fc portion of IgG (FcγRI and CD64) and FcγRI-restricted phagocytosis via TLR2 and to subvert host immunonological responses (Barrionuevo et al., 2011).

### 5.2 TLR3

It has been shown that TLR3 signaling triggered by *B. abortus* RNA contributes to cytokine responses and type I IFN expression in mouse DCs, highlighting the important role of TLR3 in proinflammatory cytokine production induced by *B. abortus* infection (Campos et al., 2017). A previous study found that *B. abortus* also down-modulates TLR3 gene and dampens the type I IFN response, leading to inefficient immune response and bacterial persistence within the host (Gorvel et al., 2014).

### 5.3 TLR4

The interaction of *Brucella* with TLR4 on host cells affects the induction of the immune response, and TLR4 plays a role in resistance to *Brucella* infection (Campos et al., 2004; Dueñas et al., 2004; Copin et al., 2007; Martirosyan et al., 2012; Ma et al., 2015; Zhu et al., 2022). *B. melitensis* OMP25 interacted with ferritin heavy polypeptide 1 (FTH1) in human placenta trophoblastic cells (HPT−8) and led to the increase of the levels of TLR4 and inflammatory factors, suggesting that OMP25 serves an important role in intracellular parasitism of *Brucella* (Zhang et al., 2022). TLR4-linked Janus kinase 2 signaling plays a pivotal role in *B. abortus* phagocytosis by macrophages (Lee, J. J. et al., 2013). The cellular oncogene c-Fos also participates in host defense mechanisms against *Brucella* infection via TLR4 signaling (Hop et al., 2018). TLR4 agonists effectively stimulate innate immunity and enhance bacterial clearance in the mouse model of brucellosis (Hedges et al., 2023).

A recent study shows that TcpB from *Brucella* interferes with the MAL-TLR4 interaction. This in turn leads to suppressing host immune responses (Sengupta et al., 2010; Radhakrishnan et al., 2011; Alaidarous et al., 2014; Saqib and Baig, 2019). *Brucella* TcpB-derived decoy peptides (TB-8 and TB-9) also inhibited TLR4 signaling and avoided host immune recognition (Ke et al., 2016). Cytoplasmic linker protein 170 (CLIP170) was found to negatively regulate TLR4-mediated proinflammatory responses by targeting TIRAP (Jakka et al., 2018). Matamoros-Recio et al. elucidated the impact of the core oligosaccharides from α2-Proteobacteria atypical lipopolysaccharides for immune system evasion in opportunistic bacteria, including *B. melitensis* (Matamoros-Recio et al., 2023). *B. abortus* O-polysaccharide (OPS) also dictates the interactions between *Brucella* and TLR4 and enhances *Brucella* persistence (Pei et al., 2008).

The modified LPS with a defective core purified from *Brucella* carrying a mutated wadC gene potentiated cytokine secretion, representing a potential for vaccine development (Conde-Álvarez et al., 2012; Conde-Álvarez et al., 2013; Zhao et al., 2018). Based on the TLR4, some groups design the multi-epitope vaccine candidates against *Brucella* (Li, M. et al., 2022; Tarrahimofrad et al., 2022; Jalal et al., 2023; Malik et al., 2023; Yin et al., 2023). *B. abortus* Omp16 lipoprotein would be able to induce a protective immune response via a TLR4-dependent manner and is a promising self-adjuvanting vaccine against brucellosis (Pasquevich et al., 2010). The enzyme lumazine synthase from *Brucella* (BLS) can insert foreign peptides and proteins at the 10 N-termini. These chimeras induced proinflammatory cytokine secretion via TLR4, providing an excellent candidate for vaccine development (Berguer et al., 2006; Berguer et al., 2012).

### 5.4 TLR5

The lack of TLR5 activity of *Brucella* flagellin is part of the stealthy strategy of *Brucella* toward the innate immune system (Terwagne et al., 2013). Hiriart et al. generated a chimeric protein by fusing flagellin from *Salmonella* in the 10 N-termini of *Brucella* lumazine synthase (BLS). This fusion protein elicits the TLR5-mediated humoral response against BLS and could be exploited as a vaccine carrier/adjuvant (Hiriart et al., 2017).

### 5.5 TLR6

TLR6 is required to trigger innate immune responses against *B. abortus in vivo* through DC maturation and proinflammatory cytokine production (de Almeida LA et al., 2013). Retamal-Díaz et al. found that TLR2/6 agonist *S*-[2,3-bispalmitoyiloxy-(2*R*)-propyl]-*R*-cysteinyl-amido-monomethoxy polyethylene glycol (BPPcysMPEG) induced improved immunogenicity and protective efficacy of a DNA vaccine encoding *B. abortus* Cu,Zn superoxide dismutase (SOD) (Retamal-Díaz et al., 2014). Woodman et al. analyzed the structural characterization of TLR1 and TLR6 from both harbor and elephant seals, identifying variants that will help to understand species-specific immune responses (Woodman et al., 2016).

### 5.6 TLR7/8

TLR7 plays an important role in IL-12 production induced by *B. abortus* infection (Campos et al., 2017). Li et al. found that overexpression of melatonin synthetic enzyme acetylserotonin *O*-methyltransferase (ASMT) enhances the resistance of transgenic sheep to brucellosis by influencing, at least in part, the TLR7 signaling pathway (Li et al., 2021). Im et al. reported that the two *B. abortus* antigens, OMP19 and malate dehydrogenase (Mdh), might be involved in the TLR8 signaling pathway in human leukemic monocyte cells (Im et al., 2018). Previous reports showed that the infection of human monocytes/macrophages with *B. abortus* inhibits the IFN-γ-induced MHC-I surface expression by a TLR8-dependent mechanism. Thus, bacteria are able to persist and establish a chronic infection inside its host (Milillo et al., 2017).

### 5.7 TLR9

Signaling pathways triggered by *Brucella* DNA involve TLR9 (Vieira et al., 2013; Campos et al., 2014; Costa Franco et al., 2018). TLR9 recognized *Brucella* CpG motifs, leading to TLR9-MAPK/NF-κB signaling pathway activation and IL-12 and TNF-α production (Gomes et al., 2016). *Brucella* DNA can be sensed by TLR9 on the endosomal membrane of macrophages and can suppress *Brucella* intracellular replication by enhancing NO production (Liu et al., 2015). Copin et al. demonstrated that the induction of IFN-gamma and inducible NO synthase (iNOS) protein induced by *Brucella* infection required TLR4 and TLR9 stimulation (Copin et al., 2007; De Trez et al., 2009). Rahimnahal et al. developed a multi-epitope vaccine against bovine brucellosis, which is capable of being in interaction with bovine TLR4 and TLR9 (Rahimnahal et al., 2023). Our group found that repetitive extragenic palindromic DNA sequences from *Brucella* stimulate TLR9 signaling (Yu et al., 2017; Peng et al., 2021).

## 6 Conclusions

In conclusion, host TLRs play a key role in the induction of the innate immune and adaptive immune responses against *Brucella* infection. The development of related drugs and vaccines is of great significance for the treatment of brucellosis. However, the process of *Brucella* infection is complex, and the fight against *Brucella* infections still faces many challenges. First, *Brucella* has evolutionarily developed diverse strategies that allow evasion of the innate and adaptive immune systems to establish persistent infections. Second, there is no effective vaccine for human brucellosis. Despite significant progress in our understanding of the molecular mechanisms of TLRs in *Brucella* infection, further focused research is needed to clarify their roles in modulating various immune events against *Brucella* pathogens.

## Author contributions

ZW: Conceptualization, Funding acquisition, Writing – original draft, Writing – review & editing. HY: Funding acquisition, Supervision, Writing – original draft, Writing – review & editing. XG: Writing – original draft. DW: Writing – original draft.
